# Therapeutic Potential of Shark Anti-ICOSL VNAR Domains is Exemplified in a Murine Model of Autoimmune Non-Infectious Uveitis

**DOI:** 10.3389/fimmu.2017.01121

**Published:** 2017-09-25

**Authors:** Marina Kovaleva, Katherine Johnson, John Steven, Caroline J. Barelle, Andrew Porter

**Affiliations:** ^1^Elasmogen Ltd., Aberdeen, United Kingdom; ^2^John Walton Muscular Dystrophy Research Centre, Institute of Genetic Medicine, Newcastle University, Newcastle, United Kingdom; ^3^Department of Molecular and Cell Biology, Institute of Medical Sciences, University of Aberdeen, Aberdeen, United Kingdom

**Keywords:** variable domain of shark new antigen receptor, single chain binding domain, shark, autoimmunity, phage display, biologic therapeutics, uveitis

## Abstract

Induced costimulatory ligand (ICOSL) plays an important role in the activation of T cells through its interaction with the inducible costimulator, ICOS. Suppression of full T cell activation can be achieved by blocking this interaction and has been shown to be an effective means of ameliorating disease in models of autoimmunity and inflammation. In this study, we demonstrated the ability of a novel class of anti-ICOSL antigen-binding single domains derived from sharks (VNARs) to effectively reduce inflammation in a murine model of non-infectious uveitis. In initial selections, specific VNARs that recognized human ICOSL were isolated from an immunized nurse shark phage display library and lead domains were identified following their performance in a series of antigen selectivity and *in vitro* bioassay screens. High potency in cell-based blocking assays suggested their potential as novel binders suitable for further therapeutic development. To test this hypothesis, surrogate anti-mouse ICOSL VNAR domains were isolated from the same phage display library and the lead VNAR clone selected *via* screening in binding and ICOS/ICOSL blocking experiments. The VNAR domain with the highest potency in cell-based blocking of ICOS/ICOSL interaction was fused to the Fc portion of human IgG1 and was tested *in vivo* in a mouse model of interphotoreceptor retinoid-binding protein-induced uveitis. The anti-mICOSL VNAR Fc, injected systemically, resulted in a marked reduction of inflammation in treated mice when compared with untreated control animals. This approach inhibited disease progression to an equivalent extent to that seen for the positive corticosteroid control, cyclosporin A, reducing both clinical and histopathological scores. These results represent the first demonstration of efficacy of a VNAR binding domain in a relevant clinical model of disease and highlight the potential of VNARs for the treatment of auto-inflammatory conditions.

## Introduction

Inflammatory eye disease, uveitis, is a significant but largely unrecognized cause of visual impairment, characterized by a very rapid and debilitating inflammation of the uvea (the pigmented and vascular structures of the eye) and requires immediate diagnosis and treatment to prevent partial or total and irreversible loss of sight. In the Western world, current incidences vary between 38 and 200 per 100,000 and it is estimated to be 730 per 100,000 in India (1). The proportion of people suffering marked visual loss may be as high as 35% with over 30,000 people in the US annually becoming blind (2, 3). Non-infectious uveitis, which is 70% of the total cases presented, is predominantly an acute manifestation of an underlying chronic autoimmune condition, and T cell activation plays a critical role in its pathogenesis (4).

There is currently no curative therapy and available treatments aim at reducing the inflammation and managing the symptoms. First-line approaches consist of corticosteroids that are often used with anti-metabolites and alkylating agents (5). For the 50% of patients who respond well, corticosteroids are inexpensive, potent and rapidly effective. However, around 30% of patients do not respond to this form of immunomodulation and the rest often suffer significant side effects, including glaucoma and cataracts, which trigger a rapid termination of therapy. Corticosteroid side effects become increasingly common as uveitis episodes recur.

When ocular inflammatory disease cannot be controlled with conventional immunosuppressive drugs (refractory patients), systemically administered adalimumab (Humira) is the most common anti-TNF-α (anti-inflammatory) agent used clinically (6). Humira completed a Phase 3 study in 2015 and reported a significant period of improvement in refractory patients from an average of 3–5.6 months and a reduction in the risk of “vision loss”. The product was approved by the FDA in 2016 for posterior uveitis (7–9). While many patients benefited from anti-TNF therapy, a significant number reported serious side effects with prolonged systemic administration considered the most probable cause of the adverse events recorded.

Shark Ig novel antigen receptors (IgNARs) are naturally occurring binding proteins known to play a role in the adaptive immune system of cartilaginous fish (10, 11). While IgNARs perform many of the duties of antibodies they have a different ancestral origin and structural architecture. IgNARs have never had, or lost, a light chain partner and have squeezed an additional binding loop into the single domain format of their two variable binding sites VNARs. An important aspect of their function is the ability to specifically bind with high affinity to target, achieved using four regions of high sequence diversity: complementarity determining region (CDR) 1, hypervariable region (HV) 2, HV4 and CDR3 (12, 13). In some species of shark non-canonical cysteine residues create an additional repertoire of VNAR isotypes that translate into structurally distinct families with diverse paratope topologies capable of binding more cryptic or hidden epitopes (13, 14). The combination of a lack of light chain partner and CDR2 makes VNARs the smallest naturally occurring binding domains in the vertebrate kingdom. This, in addition to their exquisite selectivity for target, inherent solubility and stability, makes them attractive candidates for therapeutic drug and diagnostic development (15–17).

It has been previously demonstrated by Dooley and Flajnik (18) that along with monomeric IgM, sharks can produce an antigen-specific IgNAR response following immunization. Libraries of VNARs from immunized sharks have been constructed and positive clones with high affinity and specificity to different targets like HSA, HEL, TNFα and Ebola virus have been isolated (19). In this work, phage display technology was utilized to isolate VNARs from an immunized shark library, which target and neutralize the induced costimulatory ligand (ICOSL).

ICOSL—also known as B7-related protein (B7RP-1), CD275, and B7 homolog (B7h)—is a cell surface antigen expressed constitutively on antigen-presenting cells (APCs) such as B cells, activated monocytes and dendritic cells, and is the ligand for the B7 family member, inducible costimulator (ICOS; CD278) (20). Initially, it was believed that its action was restricted to activation of T cells but more recently the central role of ICOSL in immune modulation has been expanded to both T cell stimulatory and inhibitory pathways through its interaction with CD28 and CTLA4, respectively (21). The generation of transgenic mice with lineage-restricted ICOSL expression has demonstrated the role of ICOSL–ICOS interaction in stimulating T cell responses, T cell tolerance and T cell-dependent B cell responses (22–24). The role of the ICOS/ICOSL pathway and ICOSL as a possible drug target has been previously validated in murine experimental autoimmune uveoretinitis (25). Here, we describe the isolation and characterization of VNAR domains that bind and neutralize ICOSL in a dose-dependent manner and provide the first evidence of the therapeutic potential of shark VNAR domains in a clinical model of disease. The experimental autoimmune uveitis (EAU) model of choice for this study was an interphotoreceptor retinoid-binding protein (IRBP)-induced uveitis in mice, which is considered to mirror many of the histological and clinical hallmarks of uveitis in humans (26, 27). While this study reports the benefits of systemically delivered VNAR Fc molecules, it is hoped that the progress seen here will become a stepping stone to the site-specific delivery of small, soluble and efficacious VNAR domains directly into the eye, combining the potency of biologics therapy with reduced systemic side effects.

## Materials and Methods

### Shark Immunization

Nurse sharks (*Ginglymostoma cirratum*) were placed in containers filled with artificial sea water containing 0.1% (w/v) tricaine methanesulfonate (MS-222). When the desired level of narcosis was reached, they were removed for immunization or bleeding. Recombinant in-house CHO-expressed mouse and human ICOSL-flag-His (200 μg/shark) emulsified in complete Freund’s adjuvant (CFA) were injected using a 20 gauge needle into the lateral fin of the shark. Four weeks later, antigens (200 μg/shark) emulsified in incomplete Freund’s adjuvant were similarly administered to the shark. Three immunization boosts (100 μg/shark) were given at 4-week intervals intravenously into the caudal vein as soluble antigen in PBS (sample 0.45 µM sterile filtered). Between 3 and 5 ml of blood samples were collected from the caudal vein into a 30 ml syringe containing 200 µl porcine heparin (1,000 U/ml in PBS) at weeks 0 (pre-immunization bleed), 10, 14, 18 and 22. Blood samples were spun at 1,000 × *g* for 10 min to separate blood cells from plasma. The plasma supernatant fraction was carefully removed into a sterile tube with RNA stabilization buffer and stored at −80°C.

### Serum IgNAR Titer ELISA

Immunoplates (Nunc, Thermo Scientific) were coated overnight at 4°C with 1 µg/ml human or murine ICOSL and then blocked with 4% (w/v) milk PBS (MPBS) for 2 h at 37°C. Shark serum was diluted 1:30 and then a 1:3 dilution series set up on each plate and incubated for 1 h at room temperature following incubation for 1 h with anti-nurse shark IgNAR mouse monoclonal antibody GA8 (28) (1:500 in PBST). The plates were incubated for a final time with anti-mouse IgG-HRP (SIGMA) diluted 1:1,000 in PBST. After each step, the ELISA wells were washed three times with 200 µl/well PBST. Plates were developed by adding 100 μl/well TMB substrate (Thermo Scientific) and the reaction stopped with 50 μl/well 1 M H_2_SO_4_.

### Building of a *G. cirratum* VNAR Phage Display Library

Peripheral blood lymphocytes (PBLs) were harvested from the plasma of the bleed with the best IgNAR response (titer) and total RNA isolated using a QIAGEN kit following the manufacturer’s instructions. cDNA was synthesized with IgNAR transmembrane- (Tm 5′-TACAAATGTGGTGTACAGCAT-3′) and secretory- (Sec 5′-TAGTACGACCTGAAACATTA AC-3′)-specific primers. Using the NEB Phusion HF PCR Master Mix protocol, VNAR DNA was amplified with framework (FW) nurse shark-specific primer combinations FW1/FW4r1 or FW1/FW4r2:

FW15′-GAGGAGGAGGAGAGGCCCAGGCGGCCGCTCGAGTGGACCAAACACCG-3′
FW4r15′-GAGGAGGAGGAGGAGGCCCCTGAGGCCGCATTCACAG TCACGACAGTGCCACCTC-3′
FW4r25′-GAGGAGGAGGAGGAGGCCCCTGAGGCCGCATTCACAGTCACGGCAGTGCCATCTC-3′.

Amplicons were cloned into an in-house phage display vector (pEDV1) with in-frame 6xHis-tag and c-myc-tag *via Sfi*I restriction sites. The library was transformed into electrocompetent TG1 cells (Lucigen), and the library size was calculated as described by Müller et al. (29).

### VNAR Phage Display Library Screening

To rescue phage to be used in library selections, cultures from library glycerol stocks were grown at 37°C and 250 rpm, in 2xTY, 2% glucose, 100 µg/ml ampicillin to an OD_600_ of 0.5. Cells were superinfected with 10^9^ M13K07 helper phage (NEB) and then incubated overnight in 2xTY, 100 µg/ml ampicillin, 50 µg/ml kanamycin at 25°C and 250 rpm. The cultures were PEG-precipitated (20% (w/v) PEG/2.5 M NaCl) twice, and the resulting phage pellets were resuspended in 1 ml PBS. To establish antigen “decorated” bead selections, recombinant ICOSL-flag-His protein was biotinylated with Sulfo-NHS-LC-Biotin as per manufacturer’s instructions (21327, Thermo Scientific). Two hundred microliters of magnetic Dynabeads M-280 Streptavidin (11205D, Invitrogen), pre-blocked with 2% (w/v) MPBS, were coated with 50–200 nM biotinylated material by rotating at 20 rpm, at room temperature for 1 h. Library phage was incubated first with Dynabeads for 1 h rotating at room temperature to remove phage specific to the beads and added then to the antigen-coated beads. After 1 h incubation at room temperature at 20 rpm, beads with bound phage were washed 5–10 times with PBST and 5–10 times with PBS, eluted by rotating for 8 min in 400 µl 100 mM TEA and neutralized by the addition of 200 µl 1 M Tris-HCl pH 7.5. *Escherichia coli* TG1 cells (10 ml) were infected with 300 µl of eluted phage for 30 min at 37°C and grown overnight at 37°C on TYE agar plates containing 2% (w/v) glucose and 100 µg/ml ampicillin. Two further rounds of selection were conducted, and outputs were screened for antigen-specific binding by monoclonal phage and periplasmic extract ELISAs against human or mouse ICOSL. Phage binders were detected using HRP-conjugated anti-M13 antibody (27942101, GE Healthcare), and periplasmic protein was detected using HRP-conjugated anti-c-Myc antibody (118 141 50 001, Roche).

### Expression and Purification of Monomeric VNAR and VNAR Fc-Fusion Proteins

To express monomeric VNARs, non-amber-suppressor HB2151 *E. coli* cells were used. VNARs were isolated from periplasm by osmotic shock with 50 mM Tris/HCl pH8.0, 1 mM EDTA, 20% sucrose and purified *via* His-tag capture on NiNTA resin. VNARs were eluted with 0.5 M imidazole pH 8.0 followed by dialysis in PBS. Selected positive monomeric VNAR domains were PCR amplified and subcloned into an in-house Fc-fusion mammalian expression vector (pEEE2A), which facilitated Protein A affinity purification of expressed protein post transient expression in a HEK 293 suspension culture. HEK cells at ~10^6^ cells/ml in GIBCO FreeStyle 293 media were transiently transfected using Lipofectamine 2000 (Invitrogen) according to the manufacturer’s protocol. Twenty four hours post transfection, tryptone (0.5% w/v) in PBS was added to the culture to enhance expression and the cells incubated for 5 days. Cells were pelleted at 1,000 × *g* for 15 min and the supernatants sterile filtered before adding PROSEP A resin (Millipore). After washing with PBS, fractions of purified VNAR Fc were eluted with 0.1 M glycine pH 3.0 (Severn Biotech Ltd.) and neutralized by adding 1 M Tris-HCl pH 8.0. Samples were dialyzed using Slide-A-Lyser dialysis cassettes (Thermo Scientific) in PBS pH 7.4. Expression levels of VNAR Fc-fusion proteins were generally in the range of 50–70 mg per liter using serum-free media. Electrophoresis of purified protein samples was performed on NuPAGE 4–12% Bis-Tris gels using a MES buffer system (Invitrogen) in accordance with the manufacturer’s instructions.

### Cell-Based Binding

CHO cells expressing human or mouse ICOSL were grown to 90% confluency in DMEM/F12 + 5% FBS media, in 96-well cell culture plates (Greiner, Bio-One). Anti-human or murine VNARs in DMEM/F12 + 2% FBS were added to the corresponding CHO cells. Following 1 h incubation at 16°C, cells were gently washed three times with DMEM/F12 + 2% FBS and incubated for 40 min at 16°C with anti-His-HRP (SIGMA) diluted 1:1000 in the same media. Cells were washed and developed as described previously.

### Cell-Based Ligand–Receptor Blocking Assay

CHO cells expressing human ICOS receptor were grown to 90% confluency. A total of 20 µl at 450 ng/ml of ICOSL-hFc (rhB7-H2/Fc—165-B7, R&D Systems or rmB7-H2/Fc—158-B7, R&D Systems) was preincubated for 1 h with 40 µl of serially diluted anti-ICOSL-VNAR-Fc in DMEM/F12 + 2% FBS and then added to the cells. Following 1 h incubation at 16°C, cells were gently washed three times with DMEM/F12 + 2% FBS and incubated for 40 min at 16°C with goat anti-human Fc-HRP (SIGMA) diluted 1:10,000 in the same media. Cells were washed and developed as described previously. The absorbance was measured at 450 nm wavelength using a microplate reader and data were plotted using Sigma Plot software.

### VNAR Binding ELISA

96-well flat bottom Maxisorp Nunc Immuno plates (Thermo Scientific) were coated at 4°C overnight with 1 µg/ml of the antigen of interest: for monomeric VNAR binding—ICOSL-hFc (rhB7-H2/Fc—165-B7, R&D Systems or rmB7-H2/Fc—158-B7, R&D Systems), human ICOSL-IgV, mouse ICOSL-IgV, human ICOSL-IgC, or mouse ICOSL-IgC (all ICOSL-Igs were produced in-house) and for VNAR-Fc binding—human or mouse recombinant ICOSL-flag-His (produced in-house). The plates were washed three times with 200 µl/well PBS before blocking with 200 µl of 4% (w/v) MPBS/well and incubated at 37°C for 1 h. The blocked plates were washed three times with PBS and serial dilutions of VNAR proteins were then added per designated well and the plates incubated at room temperature for 1 h. Plates were washed three times with PBST before 100 µl of 1:1,000 dilution HRP-conjugated anti-His or goat anti-human IgG antibody (for Fc-fused VNAR detection) was added to the plates and incubated for 1 h at room temperature. The plates were washed and developed by adding 100 µl TMB substrate solution and stopped using 1 M H_2_SO_4_ as previously described. The absorbance was measured at 450 nm wavelength using microplate reader and data were plotted using Sigma Plot software.

### Kinetic and Affinity Measurements of VNAR-Fc

The kinetic constants of VNAR-Fc were determined by surface plasmon resonance using Biacore T200 and 2000 biosensors (GE Healthcare). Anti-human IgG1 antibody diluted in 10 mM sodium acetate buffer was immobilized on a CM5 chip, and VNAR-Fcs were captured *via* their Fc region. HuICOSL-flag-His (100 nM) was serially diluted two-fold in HEPES running buffer (HBS EP+, BR-1006-69, GE Healthcare) with 150 mM NaCl, 3 mM EDTA and 0.05% v/v Surfactant P20 pH 7.4, and was injected for 2 min at a flow rate of 60 µl/min. Dissociation phase was monitored for 5 min followed by two 10 µl regeneration pulses using 10 mM glycine pH 1.5, at a flow rate of 100 µl/min. Association and dissociation rates were calculated using the 1:1 global Langmuir binding model fit analysis (Biacore Evaluation Software).

### Murine Model of EAU

Adult female C57BL/6 mice were randomly allocated to experimental groups and allowed to acclimatize for 1 week. Treatments were administered according to the schedule below (Table 1). Test articles were administered in PBS. On Day 0, animals were administered with an emulsion containing 500 µg of IRBP peptide 1-20 (IRBP p1-20) in CFA supplemented with 2.5 mg/ml *Mycobacterium tuberculosis* H37 Ra by subcutaneous injection. Also on Day 0, animals were administered with 1.5 µg *Bordetella pertussis* toxin by intraperitoneal injection.

**Table 1:** Schedule of *in vivo* treatments.

Group	Treatment	Dose	Route	Frequency	Disease induction
1	Untreated	n/a			
2	Cyclosporin A	20 mg/kg	PO	SID, Day 1–Day 28	Day 0: IRBP/CFA, SC
3	A5-Fc	10 mg/kg	IP	SID, Day 1–Day 14	Day 0: PTx, IP

*n/a, not applicable; PO, oral administration; SC, subcutaneous injection; IP, intraperitoneal injection; SID, once daily; IRBP, interphotoreceptor binding protein; CFA, complete Freund’s adjuvant; PTx, pertussis toxin*.

From Day 7 until the end of the experiment on Day 28, animals were monitored once per week for clinical signs of uveitis using topical endoscopic fundal imaging (TEFI) (Table 2). Animals were also monitored twice weekly for signs of ill-health, weighed and any abnormalities recorded. At termination on Day 28, eyes were removed into tissue fixative for histopathology.

**Table 2:** Clinical score ranking using topical endoscopic fundal imaging.

Clinical score	Optic disk inflammation	Retinal vessels	Retinal tissue infiltration	Structural damage
1	Minimal	1–4 mild cuffings	1–4 small lesions or 1 linear lesion	Retinal lesions or atrophy involving 1/4–3/4 of retinal area
2	Mild	>4 mild cuffings or 1–3 moderate cuffings	5–10 small lesions or 2–3 linear lesions	Panretinal atrophy with multiple small lesions (scars) or ≤3 linear lesions (scars)
3	Moderate	>3 moderate cuffings	>10 small lesions or >3 linear lesions	Panretinal atrophy with >3 linear lesions or confluent lesions (scars)
4	Severe	>1 severe cuffings	Linear lesion confluent	Retinal detachment with folding

5	Not visible (white-out or severe detachment)			

### Corneal Penetration

Wild-type mice BALB/C were divided into three groups of two animals each. All procedures were performed on anesthetized animals. Corneal epithelium of the right eye was scratched and 20 µg/3 μl of VNAR, VNAR Fc, or mAb AF158 (R&D) was applied four times topically to the right eye at 5-minute intervals. The eye was then washed with saline solution and 2 µl of anterior fluid sampled. All animal studies were carried out under the Animals (Scientific Procedures) Act 1986 regulations (Home Office UK). For ELISA, 96-well flat bottom Maxisorp Nunc Immuno plates (Thermo Scientific) were coated with 1 µg/ml of rmB7-H2/Fc or mICOSL-flag-His at 4°C overnight. The plates were washed three times with 200 µl/well PBS before blocking with 200 µl of 4% (w/v) MPBS per well and incubated at 37°C for 1 h. The blocked plates were washed three times with PBS, 100 µl of anterior fluid (start dilution 1:50 in PBS, then serial dilutions 1:2) was then added and incubated at room temperature for 1 h. Plates were washed three times with PBST and 100 µl of 1:1,000 dilution HRP-conjugated anti-His (for VNAR detection) or goat anti-human IgG antibody (for Fc-fused VNAR and mAb detection) was added to the plate and incubated for 1 h at room temperature. The plates were washed and developed by adding 100 µl TMB substrate solution and stopped using 1 M H_2_SO_4_ as previously described.

## Results

### VNAR Library Construction from Immunized Nurse Shark

Two nurse sharks were immunized with both recombinant human and mouse ICOSL. An antigen-specific IgNAR immune response was observed and confirmed after 18 weeks through the analysis of post-immunized sera. The response was exemplified by a gradual increase in titer values from early to late bleeds (data not shown). The VNAR repertoire was amplified from isolated PBLs, cloned into a pEDV1 which contained an in-frame coat protein pIII of the bacteriophage M13 gene and transformed into *E. coli* TG1 cells. The library size was calculated to be 10^8^ transformants.

### Isolation of Human ICOSL-Specific VNARs

**Figure 1:**
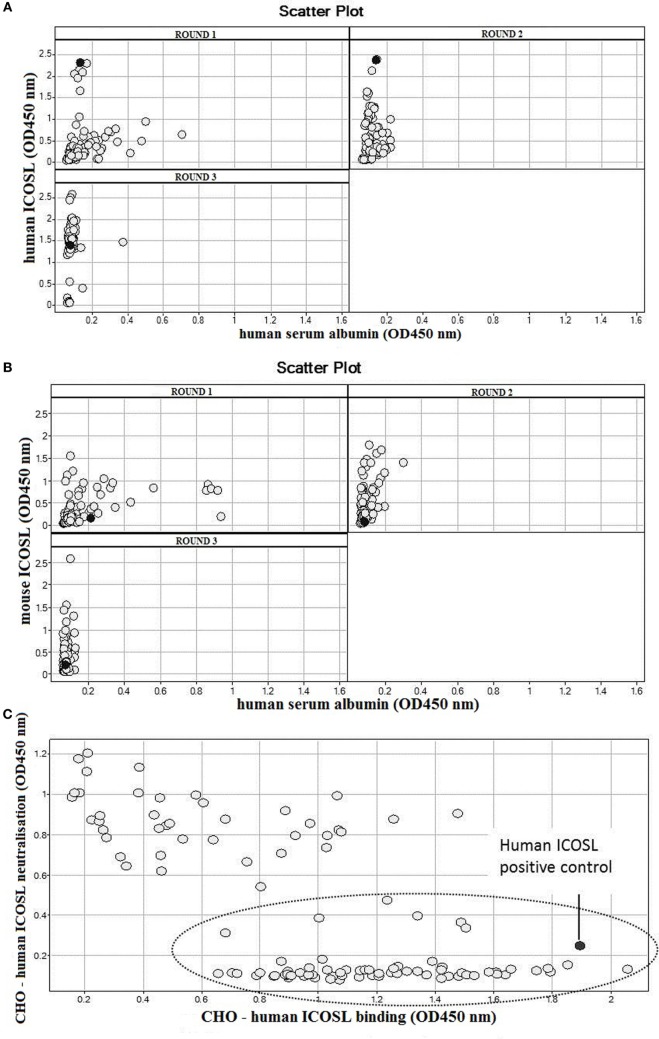
Selection of anti-ICOSL VNARs. **(A)** Screening of outputs from a selection campaign with human ICOSL. The scatter plot represents screening data of phage monoclonals from each round of selection for specific binding to huICOSL (*Y* axis) vs non-specific binding to human serum albumin (*X* axis). Each circle denotes a single clone. **(B)** Screening of outputs from a selection campaign with mouse ICOSL. The scatter plot represents screening data of phage monoclonals from each round of selection for specific binding to mICOSL (*Y* axis) vs non-specific binding to human serum albumin (*X* axis). **(C)** Cell-based binding and huICOS-huICOSL blocking assay. Monoclonal VNAR outputs from third round of selection with huICOSL were expressed in periplasm, and periplasmic fractions were tested in cell-based binding and ICOS-ICOSL blocking assays. The *X* axis indicates CHO-huICOSL binding with higher signals corresponding to stronger binders and the *Y* axis identifies clones with decreased signals that are capable of blocking the interaction of ICOSL with CHO-huICOS. The circled area captures all clones which are both strong huICOSL binders and can block ICOS/ICOSL interaction. The human ICOSL positive control is the mouse monoclonal anti-huB7-H2 antibody (MAB165, R&D).

**Figure 2:**
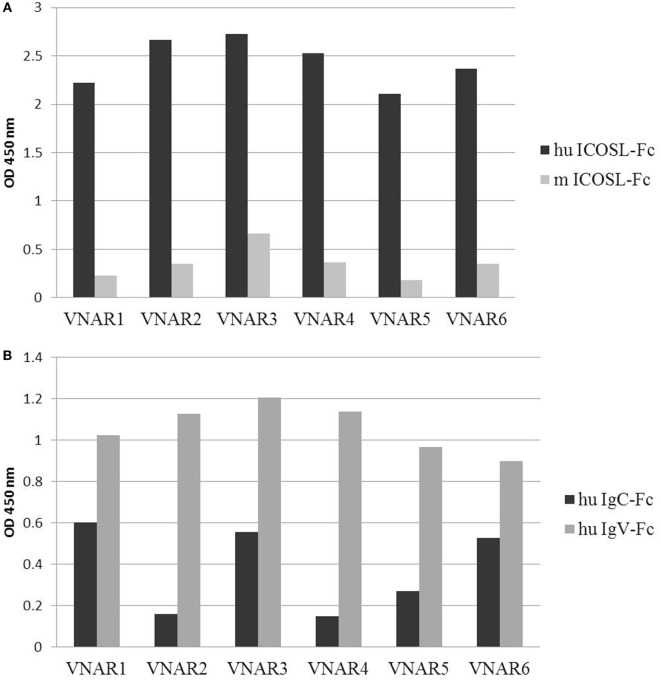
Characterization of anti-huICOSL lead domains binding. **(A)** Lead anti-huICOSL VNAR domains were tested for binding to human and mouse ICOSL in ELISA. **(B)** Binding of lead clones to the IgC and/or IgV domain of the ICOS ligand.

Domains were isolated following three rounds of selection utilizing biotinylated antigen immobilized on streptavidin-coated beads to maximize the chance of delivering potent ICOSL binders (30). huICOSL-specific VNARs were obtained after the first selection round and enriched further after round three with >90% of domains specific for antigen (Figure 1A). As blockade of the ICOS–ICOSL interaction was the desired functional outcome of the selection process, a cell-based assay designed to detect domains that can block receptor/ligand binding was introduced into the screen. Positive hits from selections against huICOSL were assessed for their ability to block huICOSL binding to ICOS expressing cells (Figure 1C). Signals that decreased by 50% or more were considered positive for receptor–ligand binding inhibition. In total, six unique (based on CDR3 sequence differentiation) anti-human ICOSL VNAR clones were identified. Cell surface antigen-target selectivity was assessed by FACS analysis utilizing CHO cells overexpressing human or murine ICOSL (data not shown) as well as in a binding ELISA format (Figure 2A). All domains were found to be strong huICOSL binders but with no species cross-reactivity to the mouse ligand. The affinity of huICOSL binders was in the range 1–9 nM (Table 3). ICOSL is a two-domain protein, where receptor binding is mediated solely by the membrane distal IgV domain but requires the membrane proximal IgC domain to maintain the structural integrity of the protein (31). All of the isolated blocking huICOSL VNARs cross-reacted strongly with the ICOSL-IgV domain with weaker or negligible binding to the ICOSL-IgC domain (Figure 2B). As the percentage homology between the human and murine IgV region of ICOSL is only 43%, it was not surprising that the screen did not isolate species cross-reactive VNARs that block receptor/ligand interaction.

**Table 3:** Kinetic parameters and affinity determination of VNAR-Fcs binding to human ICOSL.

VNAR-Fc	ka (1/Ms)	kd (1/s)	KD (M)
VNAR 1	3.11 × 10^5^	9.51 × 10^−4^	3.06 × 10^−9^
VNAR 2	5.50 × 10^5^	6.69 × 10^−4^	1.22 × 10^−9^
VNAR 3	5.96 × 10^5^	1.00 × 10^−3^	1.68 × 10^−9^
VNAR 4	8.73 × 10^5^	3.93 × 10^−3^	4.50 × 10^−9^
VNAR 5	3.73 × 10^5^	4.15 × 10^−3^	1.11 × 10^−8^
VNAR 6	3.26 × 10^5^	2.73 × 10^−3^	8.39 × 10^−9^
Isotype control 2V			Did not bind

*Kinetic measurements of anti-huICOSL domains. Kinetic analysis of six anti-huICOSL lead domains is summarized in the table. The interaction between the lead clones and huICOSL was measured by surface plasmon resonance (SPR). An anti-human IgG antibody was immobilized on a Biacore chip and the VNAR clones captured *via* their Fc region*.

*ka—association rate constant; kd—dissociation rate constant; KD—binding affinity*.

### Isolation of Mouse ICOSL-Specific VNARs

**Figure 3:**
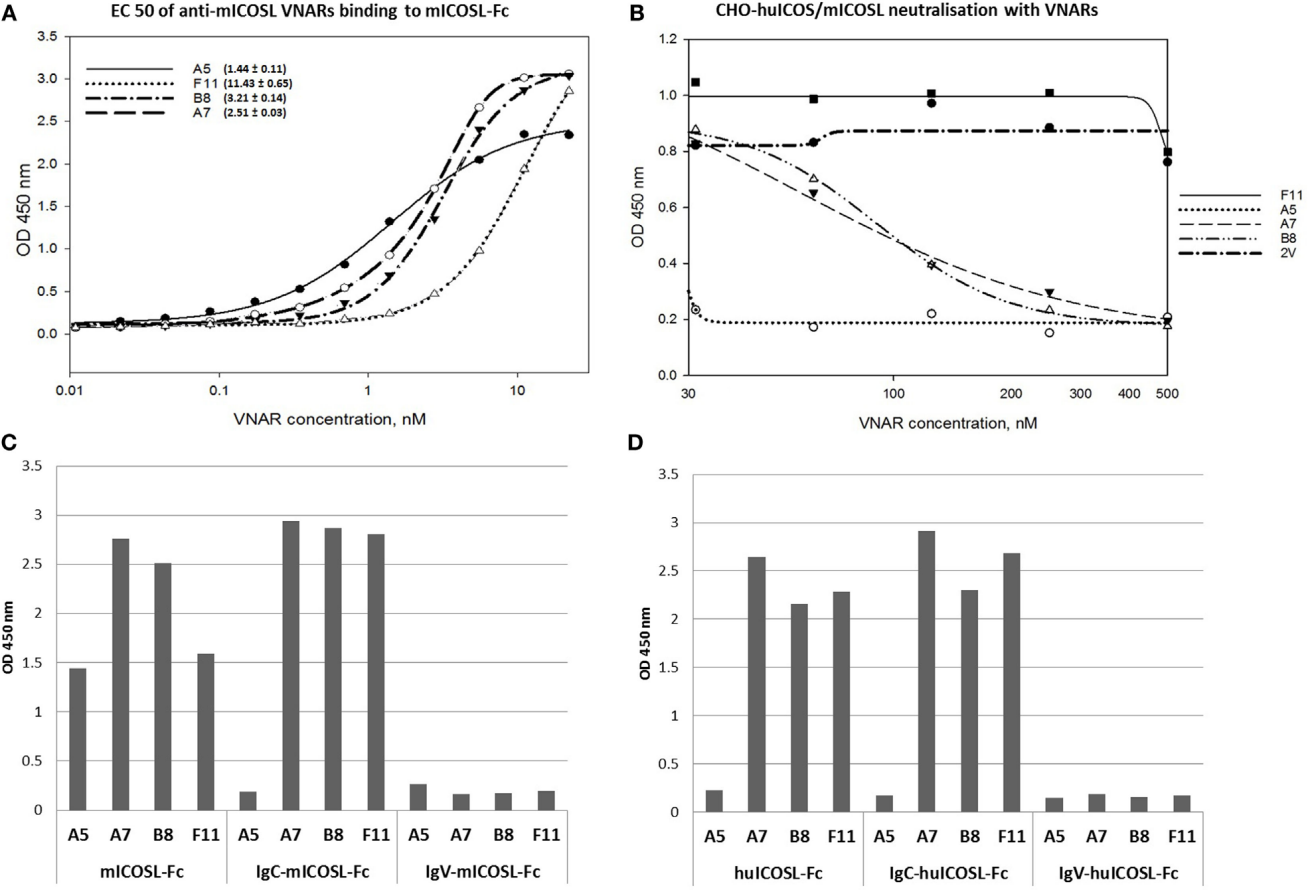
Binding specificity of anti-mICOSL lead domains and blocking of ligand/receptor interaction. **(A)** Binding to mouse ICOSL. Titration curves of four lead anti-mICOSL domains binding to recombinant mouse ICOSL in ELISA and calculated EC50 values. **(B)** Blocking of ligand/receptor binding. Concentration-dependent inhibition of mICOSL-Fc binding to cell surface expressed hICOS by the addition of serial dilutions of anti-mICOSL VNAR domains (from 30 to 500 nM). 2V is the VNAR isotype control used in this experiment. **(C)** Binding to the IgC and IgV domains of the mouse ICOSL. **(D)** Binding to IgC and IgV part of the human ICOSL.

Anti-mouse ICOSL VNAR domains were isolated using the same method as for the anti-human domains and lead clone isolation determined following their performance in *in vitro* (Figure 1B) and cell-based binding assays. Four unique binders, all within an EC50 (effective concentration) range from 1.4 to 11.4 nM (Figure 3A), were taken forward for further study. Three of these anti-mICOSL clones (A5, A7, and B8) could block ligand/receptor binding in a CHO-cell-based blocking assay, whereas clone F11 lacks this blocking activity and was used here as a non-blocking control (Figure 3B). The naïve VNAR domain 2V was also included as an isotype control in ligand/receptor binding assays. This clone originally isolated from the dogfish (*Squalus acanthias*) is part of a sequence database from this species and has no known target, making it an ideal negative control for these and other studies (11).

### Specificity of Anti-mICOSL VNARs

Unlike VNARs obtained from the human ICOSL selection campaign, domains isolated from mouse ICOSL selections were species cross-reactive. Three of the four clones recognize both mouse and human ligand in an ELISA with clear binding to IgC domains (Figures 3C,D). Interestingly, clone A5, which was the strongest ICOS/ICOSL blocker (Figure 3B), bound only to the full length mouse protein, but not to the individual IgV and IgC domains implying that it may recognize an interdomain or linking region between them (Figure 3C).

### Fc-Reformatting of Anti-mICOSL VNARs

**Figure 4:**
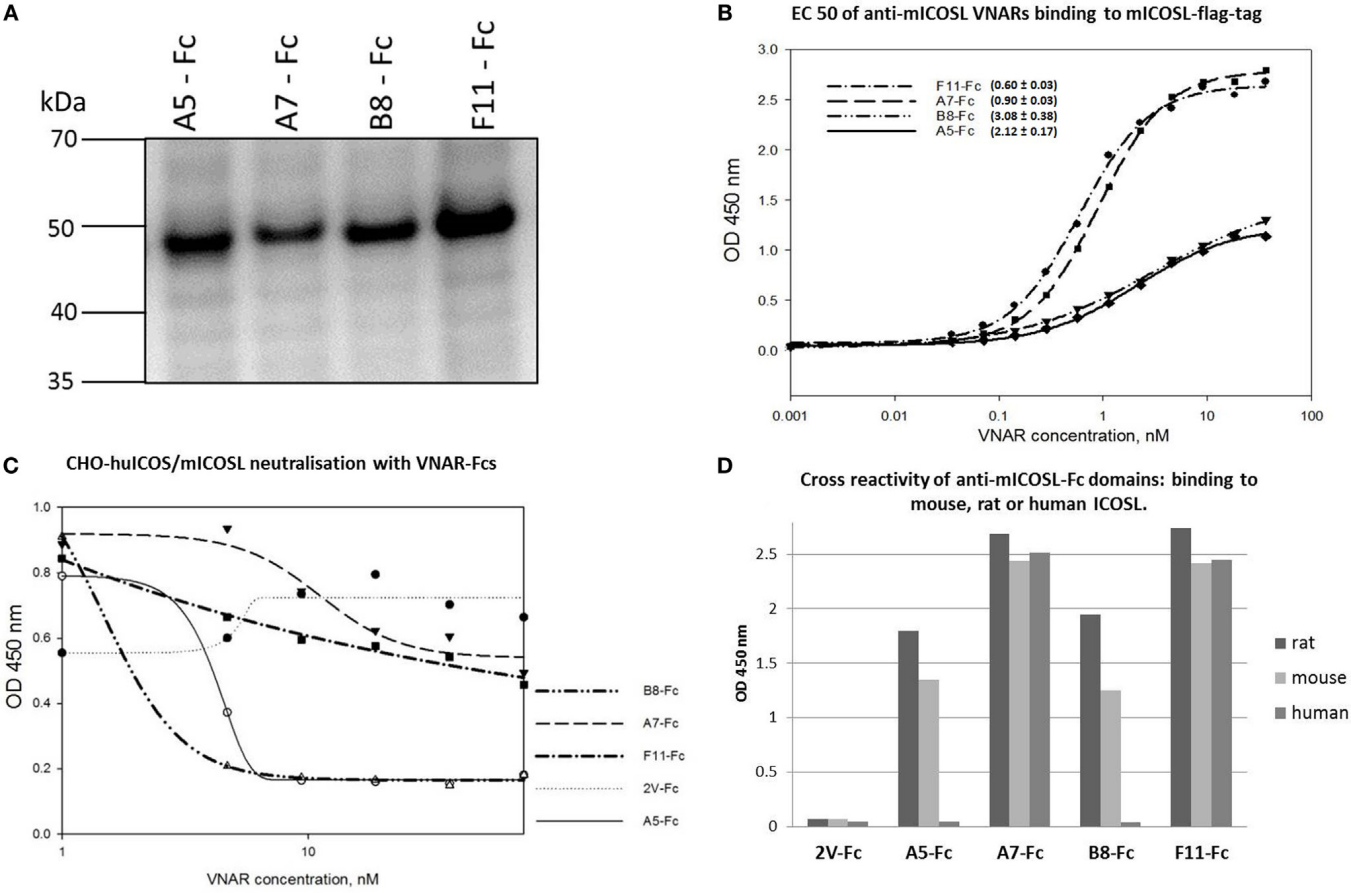
Characterization of anti-mICOSL lead domains after Fc reformatting. **(A)** SDS-PAGE of HEK293 expressed VNAR Fc. **(B)** Binding to mouse ICOSL. Titration curves of anti-mICOSL-Fc domains binding to recombinant mouse ICOSL. **(C)** Blocking of ligand/receptor binding. Concentration-dependent inhibition of recombinant mICOSL-Fc binding to cell surface expressed hICOS by the addition of serial dilutions of anti-mICOSL-Fc domains. 2V-Fc is the isotype control. **(D)** Species cross-reactivity of anti-mICOSL-Fc domains.

A key aim of this work was to determine the efficacy of VNAR domains in an *in vivo* mouse model. As VNAR domains alone are cleared rapidly from the systemic circulation (11), all VNAR clones were first converted into a fusion format with a human Fc (Figure 4A) to facilitate an extension of serum half-life. All VNAR Fcs retained binding to mICOSL with improved, presumably through avidity, EC50s in the range 0.6 to 3 nM (Figure 4B). In cell-based ICOS/ICOSL blocking assays conducted with Fc-reformatted VNAR domains, clone A7, which performed well as a monomeric VNAR, lost this ability after Fc-fusion (Figure 4C). The reason for the loss of activity was not investigated further at this time. In summary, A5-Fc and B8-Fc, both blocked receptor/ligand interaction, did not bind human ICOS ligand but did bind mouse and rat protein. F11-Fc did not block ICOS/ICOSL binding, and A7-Fc, which lost its blocking ability after reformatting, bound ligands from all three species (Figure 4D).

### Evaluation of Anti-mICOSL VNARs in a Murine Model of EAU

**Figure 5:**
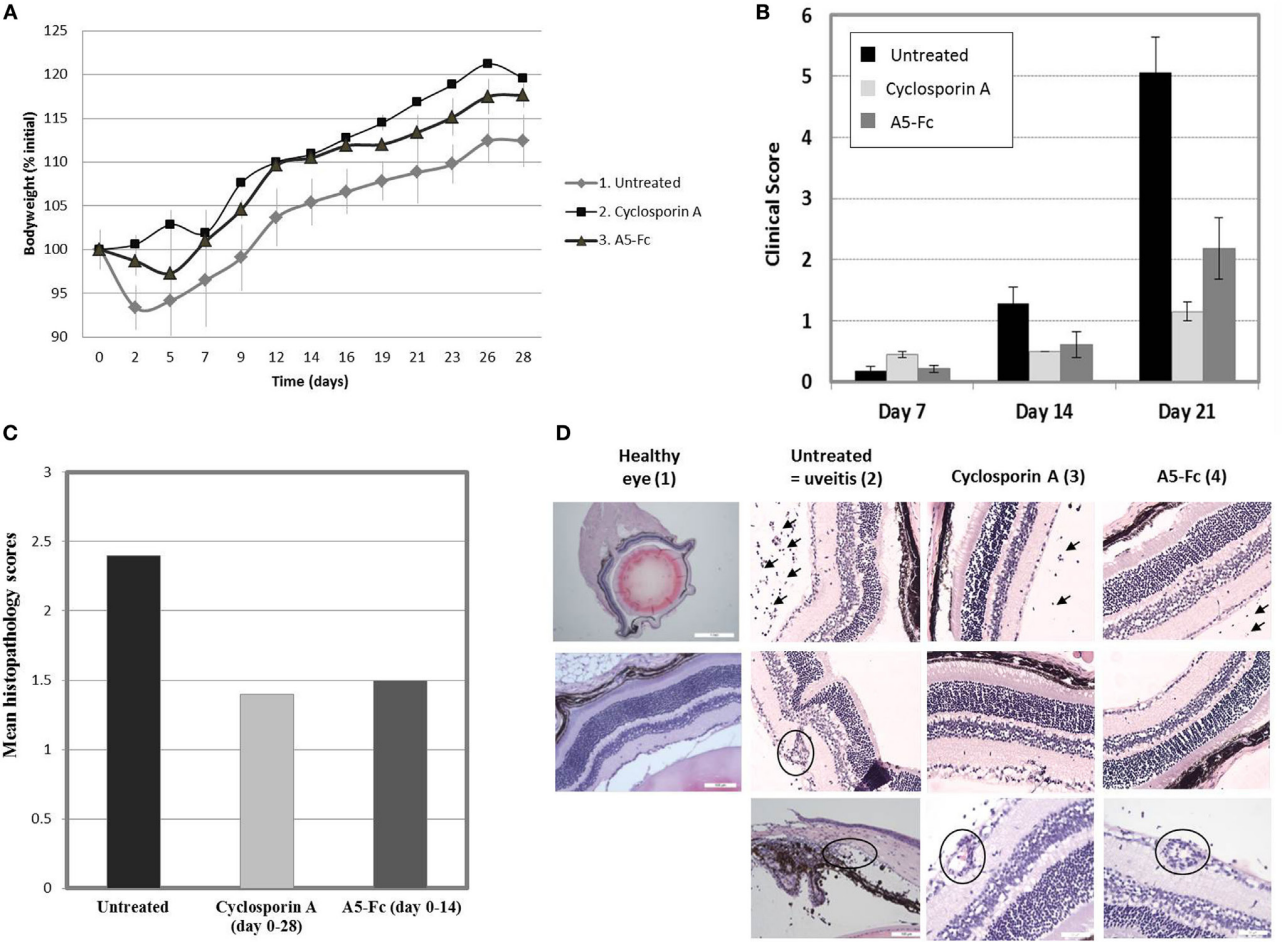
Clinical scores and histopathology sections from the interphotoreceptor retinoid-binding protein-induced uveitis study. **(A)** Bodyweights. All animals were weighed three times a week. Data are presented as mean ± SEM of percentage initial (Day 0) bodyweights. No bodyweight loss was observed. **(B)** Clinical scores. Retinal imaging by topical endoscopic fundal imaging (TEFI) was analyzed by one-way ANOVA followed by Dunn’s test for multiple comparisons between experimental days. A significant increase in TEFI scores was observed on Day 21 when compared to Day 7 in the untreated group, as expected for this model of uveitis. Cyclosporin A, administered from Day 1 until the end of the experiment, induced a significant reduction in the clinical scores when compared to the untreated group at Day 21. A5-Fc, administered from Day 1 until Day 14 of the experiment, delivered a comparable result to the Cyclosporin A group treated for 28 days. **(C)** Histopathology scores. Histopathology scores were analyzed by the Kruskal–Wallis test followed by Dunn’s post-test for multiple comparisons between experimental groups. The pathological changes observed were consistent with those reported for the model. Greater inflammation was observed in Group 1 (untreated). Cyclosporin A administered from Day 1 until Day 28 and A5-Fc administered from Day 1 until Day 14 caused an equivalent reduction in the histopathology scores. **(D)** Histopathology sections. Dissected eyes were embedded in paraffin wax, sectioned, and stained with hematoxylin and eosin for detailed histopathology analysis at the cellular level. (1) Healthy entire eye glob section and normal retina. (2) Untreated (=uveitis): inflammatory cells in the vitreous (upper panel), a cuff of inflammatory cells surrounding the vessel (middle panel) and neutrophilic inflammation in the drainage angle, anterior chamber, and ciliary body (lower panel). (3) Cyclosporin A treated: mild inflammation of the vitreous (upper and middle panels) and mild vasculitis (lower panel). (4) A5-Fc treated: a low number of inflammatory cells (mild inflammation) in the vitreous (above and middle panels) and mild vasculitis/cuffing (lower panel).

The lead domain A5-Fc was assessed in a murine model of EAU by KWS (Bristol, UK). Adult female C57BL/6 mice were randomly allocated to experimental groups and allowed to acclimatize for 1 week. Treatments were administered according to the protocol described in Section “Materials and Methods” from Day 1 to Day 14 or Day 1 to Day 28 for the corticosteroid control (five mice per group) and from Day 1 to Day 14 for the A5-Fc protein (six mice per group). On Day 0, animals were administered with IRBP p1-20 in CFA supplemented with *M. tuberculosis* H37 Ra to induce uveitis. All animals were weighed three times a week and also monitored twice weekly for signs of ill-health and any abnormalities recorded. The disease-induction procedure (subcutaneous administration of IRBP/CFA and intraperitoneal injections of pertussis toxin) caused a mild bodyweight loss on Day 2 and Day 5, as expected for this model. However, from day 7 until the end of the experiment, there was no further treatment-induced bodyweight loss in any of the experimental groups (Figure 5A). From day 7 until the end of the experiment, animals were monitored once a week for clinical signs of uveitis using TEFI. A significant increase in TEFI scores was observed on Day 21 when compared with the untreated Day 7 control group. By day 21, both the Cyclosporin A and the anti-mICOSL VNAR domain A5-Fc groups showed a significant reduction in recorded clinical scores and a marked lag in the onset of any disease when compared to the untreated control animals (Figure 5B). Histopathology analyses of eyes, removed at the end of the experimental period (Day 28), confirmed that this observed reduction in inflammation translated into differences at the tissue level too (Figure 5C). Furthermore, detailed cellular observation clearly showed that animals treated with either Cyclosporin A or with anti-mICOSL VNAR domain A5-Fc presented with mild disease pathology compared to the untreated control. The control animals exhibited extensive cellular inflammation with a high number of neutrophilic cells accumulating in the vitreous, the drainage angle, the anterior chamber, the ciliary body and also in the surrounding blood vessels (Figure 5D).

### Corneal Penetration of VNAR

**Figure 6:**
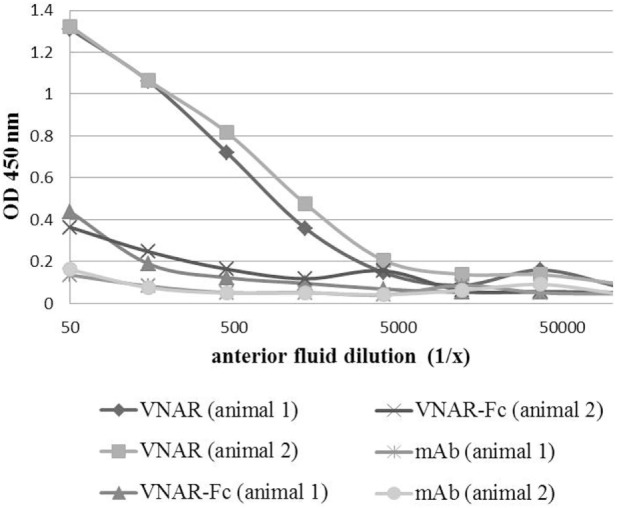
Corneal penetration of VNAR, VNAR Fc, and mAb. A mouse eye with a scratched cornea (to remove epithelium) was treated for 20 min with VNAR (two animals), VNAR-Fc (two animals), or mAb (two animals) by applying drops (4 × 3 µl) directly onto the eye. Anterior fluids were collected and analyzed in ELISA for the presence of VNAR, VNAR Fc, or mAb. VNAR, but not mAb or VNAR-Fc, was detected in anterior fluid of both mice.

The influence of molecular size on the ability of biologics to enter the eye if applied topically was assessed in a scratched corneal mouse model that goes some way to mimicking the situation seen in severe inflammatory eye conditions. It was hoped that the smaller size of the VNAR domains may provide a delivery option for site-specific targeting of ocular disorders. Three formats of increasing molecular weight were tested: VNAR single domains (11 kDa), VNAR Fc (80 kDa) and mAb (150 kDa). All three were applied dropwise onto cornea without an epithelium and anterior fluid was collected after 20 min of treatment. The presence of VNAR was clearly observed in the anterior fluid compared to VNAR-Fc and mAb (Figure 6).

## Discussion

Uveitis, a major cause of severe visual loss around the world, may be idiopathic or occur as a part of systemic disease such as spondyloarthritis, Vogt–Koyanagi–Harada syndrome, sarcoidosis, autoimmune hepatitis, systemic lupus erythematosus and multiple sclerosis (32). First-line therapy for patients with active uveitis is corticosteroids because of their rapid effect and the flexibility in the choice of their delivery—locally to the eye or systemically. However, long-term corticosteroid treatment is associated with the risk of various adverse events including cataract, glaucoma, diabetes, cushingoid changes, hypercholesterolemia and osteoporosis (33). If a desired response is not achieved with short-term therapy and/or corticosteroids fail to control the inflammation, biological agents may be required for the treatment of worsening or refractory disease. Immunomodulatory biologics are powerful drugs that have been used to treat immune-related diseases in a number of different therapeutic areas. They can be designed to dampen down hyperimmune responses and therefore have utility in chronic autoimmune and inflammatory conditions (21). However, it is well known that the prolonged use of powerful systemic biologics can also result in the development of significant off-target side effects and patient complications.

Like the development of many drugs, biologics often rely upon animal models as predictors of clinical efficacy. However, due to the inherent high target specificity of biologics and in some cases low target homology across species, the use of rodent models for preclinical efficacy and safety studies is sometimes precluded. When this situation occurs it is common practice to develop a surrogate or analogous candidate (e.g., for anti-TNF clinical biologics development) that targets the orthologous protein in rat or mouse (34–38). Here, anti-mICOSL VNAR domains could be considered surrogates for the use of anti-hICOSL VNARs in patient therapy. In this study, we have isolated anti-mouse and anti-human VNARs from a nurse shark immunized library that can inhibit the receptor/ligand interaction and demonstrated for the first time the efficacy of an anti-mICOSL VNAR domain in a clinically relevant mouse model of EAU. The EAU model is well characterized and widely used as a clinical model of human non-infectious uveitis (39, 40). ICOSL and its importance in antibody-mediated human disease have been verified in a number of preclinical models including RA, SLE and uveitis (22–24, 41–44). The effectiveness of using a mouse mAb to block the interaction between ICOS-ICOSL has also previously been demonstrated in EAU (43).

Shark VNAR domains have been previously isolated against a number of targets from both semisynthetic and immunized sources (10, 11, 19, 45–52). The first demonstration of *in vivo* VNAR activity showed that an anti-HSA VNAR domain isolated from an immunized dogfish could extend the serum half-life of a fused partner VNAR across mouse, rat and monkey pharmacokinetic models (11). In later work, a single anti-TNFα VNAR domain was isolated from an immunized horn shark and showed a modest level of inhibition in a murine model of endotoxic shock (53). Most recently, the isolation of B cell-activating factor-specific VNAR from a synthetic library was shown to function as selective B-cell inhibitors to target B-cell disorders (52). However to date, this uveitis study is the first example of a VNAR domain showing significant *in vivo* efficacy in a recognized clinical model, establishing the potential for the future development of human-specific surrogates as effective treatments of autoimmune disease.

Current advances in ocular drug delivery technologies, suitable for the administration of smaller molecular weight biologics (54–56), provide an encouraging future for the use of VNARs (or their humanized equivalents) in ophthalmology and offer the promise of effective site-specific and systemic side effect-free delivery. At 11 kDa, VNARs are the smallest domains of their type in the animal kingdom and are almost 13 times smaller than an antibody, making the delivery of VNAR to the back of the eye more achievable. We have presented here preliminary evidence that VNAR, if applied topically and at high concentrations, can cross the cornea and be found in the anterior chamber of a mouse eye, whereas an mAb or VNAR-Fc could not. The amounts delivered appeared to reflect the molecular weight of the formats tested with VNAR Fc observed in anterior fluid but at much lower concentrations compared to the single VNAR. It is therefore attractive to speculate that combining the benefits of VNAR domains with a validated inflammatory disease target and new drug delivery technologies could result in the development of a viable drug candidate for ocular disease treatment that could be administered either systemically and/or site-specifically.

## Ethics Statement

This study was carried out in accordance with the recommendations of HMRC under the project license—PPL 7008395. The protocol was approved by the College of Life Sciences and Medicine Ethics Review Board. Work conducted by KWS BioTest (Registered Address: 4 Clifton Road, Clifton, Bristol, BS8 1AG. Registered No. 4980013) was under their ethical guidelines and authorized animal procedures.

## Author Contributions

CB—leads the science team at Elasmogen and was colead on this program of work alongside AP. Both edited the manuscript. MK—scientific lead on this paper who carried out the majority of the scientific work and wrote the manuscript. KJ—conducted the isolation of the immunized clones and initial characterization and edited the paper. JS—led the expression and purification of the clones.

## Conflict of Interest Statement

MK, JS, CB and AP are affiliated with Elasmogen Ltd. All other authors have no competing interests to disclose. The reviewer MR and handling Editor declared their shared affiliation.

## Abbreviations

IgNAR, immunoglobulin like new antigen receptor; VNAR, variable domain of shark new antigen receptor; ICOSL, induced costimulatory ligand; CDR, complementarity determining region; FW, framework; HV, hypervariable region; IRBP, interphotoreceptor retinoid-binding protein; EAU, experimental autoimmune uveitis; APC, antigen-presenting cell.
